# Prevalence and Genetic Diversity of Avian Haemosporidian Parasites in Southern Iran

**DOI:** 10.3390/pathogens10060645

**Published:** 2021-05-23

**Authors:** Vajiheh Ghaemitalab, Omid Mirshamsi, Gediminas Valkiūnas, Mansour Aliabadian

**Affiliations:** 1 Department of Biology, Faculty of Science, Ferdowsi University of Mashhad, Mashhad 9177948974, Iran; ghaemitalab.vajihe@mail.um.ac.ir (V.G.); mirshams@um.ac.ir (O.M.); 2 Research Department of Zoological Innovations (RDZI), Institute of Applied Zoology, Faculty of Science, Ferdowsi University of Mashhad, Mashhad 9177948974, Iran; 3 Nature Research Centre, Akademijos 2, 08412 Vilnius, Lithuania; gediminas.valkiunas@gamtc.lt

**Keywords:** birds, haemosporidian parasites, *Plasmodium*, *Haemoproteus*, *Leucocytozoon*, Iran

## Abstract

Avian haemosporidians are widespread and diverse and are classified in the genera *Plasmodium*, *Haemoproteus, Leucocytozoon*, and *Fallisia*. These species are known to cause haemosporidiosis and decreased fitness of their hosts. Despite the high diversity of habitats and animal species in Iran, only few studies have addressed avian haemosporidians in this geographic area. This study was performed in the south and southeast of Iran during the bird breeding seasons in 2017 and 2018, with the aim to partly fill in this gap. Blood samples of 237 passerine birds belonging to 41 species and 20 families were collected. Parasite infections were identified using a nested PCR protocol targeting a 479-base-pair fragment of the mitochondrial cytochrome *b* (*cytb*) gene of *Haemoproteus*, *Plasmodium* and *Leucocytozoon* species. The overall prevalence of haemosporidian parasites was 51.1%, and 55 different lineages were identified, of which 15 *cytb* lineages were new globally. The lineages of *Haemoproteus* predominated (63.6% of all detected lineages), followed by *Leucocytozoon* and *Plasmodium*. Nineteen new host records of haemosporidian *cytb* lineages were identified, and the majority of them were found in resident bird species, indicating local transmission. Thirteen co-infections (9.8% of infected individuals) of *Haemoproteus* and *Leucocytozoon* parasites in seven host species were observed. This study shows the presence of active local transmission of parasites to resident bird species in the southeast of Iran and contributes to the knowledge on haemosporidian parasite biodiversity in this poorly studied region of the world.

## 1. Introduction

Haemosporidian parasites (Haemosporida) are widespread intracellular pathogens that infect many species of terrestrial vertebrates including birds, mammals and reptiles [1,2,3,4]. Avian haemosporidians is the largest group of haemosporidians in terms of number of described species and an excellent model for the study of host–parasite interactions [1,5]. The knowledge on prevalence, diversity and patterns of distribution of avian haemosporidians can be useful for better understanding various ecological, evolutionary, and behavioural questions [4], as well as important in conservation biology and disease risk assessment in veterinary medicine and wildlife populations [2,6,7]. This is particularly true for co-infections with two or more different haemosporidian parasites, which could lead to disease, accompanied by anaemia, loss of body mass and reduced survival in hosts [8,9].

The haemosporidian parasites of birds belong to the genera *Plasmodium* Marchiafava and Celli 1885, *Haemoproteus* Kruse 1890*, Leucocytozoon* Berestneff 1904 and *Fallisia* Lainson, Landau and Shaw 1974, which have been detected all around the world except in Antarctica [1,10]. *Fallisia* parasites were found in birds only in South America. Since the development of molecular identification of avian haemosporidians using fragments of the mitochondrial cytochrome *b* gene [11,12], the number of scientific publications, parasite lineages and bird taxa studied has remarkably increased [13,14]. These findings have revealed much higher than expected levels of parasites diversity [7]. Based on the MalAvi database (a Public Database of Malaria Parasites and Related Haemosporidians in Avian Hosts, www.mbio-serv2.mbioekol.lu.se, accessed on 1 May 2021), over 3600 unique haemosporidian lineages have been reported in about 20% of bird species worldwide. Furthermore, co-infection with two or more different haemosporidian parasites was shown to be common in wild birds [6,15,16,17]. 

Iran is an important area of bird biodiversity [18]. Its territory was classified in four eco-zones, located in the north (eastern and western Palearctic), south (Afrotropical) and southeast (Oriental) of the country [19]. These geographical areas are characterized not only by markedly different habitats but also by bird species diversity [20]. However, information about malaria and related haemosporidian parasite diversity remains insufficiently studied in Iran, and this is an obstacle for better understanding these parasite biodiversity patterns in southern Asia. In recent years, several studies have addressed avian haemosporidians, mainly in domestic and aquatic birds in Iran [21,22,23,24,25,26,27,28,29]. Nevertheless, a few studies have been conducted on haemosporidians of songbirds [30,31].

The order Passeriformes (passerines) includes more than 60% of described bird species and is the largest and most species-diverse avian order [32]. It includes 230 species belonging to 32 families inhabiting diverse habitats in Iran. About 110 passerine species are residents or summer breeding in southern parts of the country [33]. However, few studies examined their blood parasites in this region. Resident non-migrating bird species are of particular interest for parasitology research, because their parasite fauna develops locally and is an indication of local parasite transmission. This study aimed to identify the *cytb* lineages of *Plasmodium*, *Haemoproteus* and *Leucocytozoon* in passerines over the southwest to the southeast of Iran in six provinces and to determine the prevalence and genetic diversity of these pathogens. This knowledge is important for a better understanding of the epidemiological situation regarding haemosporidiosis in Iran and will also contribute with valuable data to the description of the biogeography and biodiversity of avian haemosporidians.

## 2. Results

### 2.1. Parasite Prevalence

**Figure 1:**
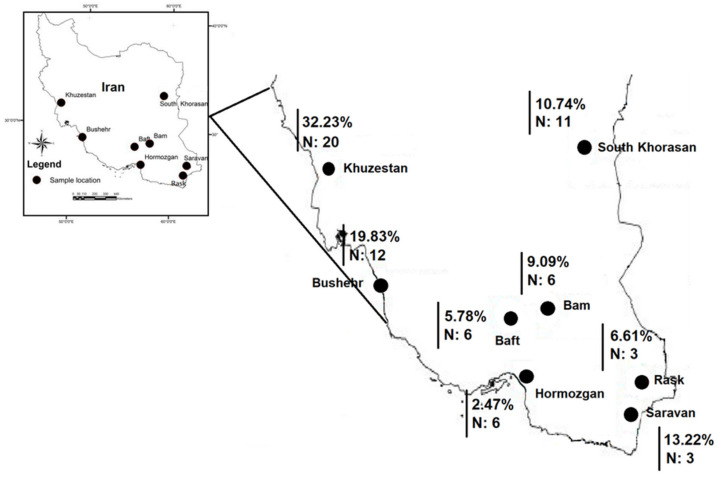
The localities in southern Iran where birds were examined. Sampling localities (black dots) are Khuzestan, Bushehr, Hormozgan, South Khorasan provinces, Rask (Sistan and Baluchistan province), Saravan (Sistan and Baluchistan province), Bam (Kerman province), Baft (Kerman province). Prevalence of infection (in percentage) is shown in each locality. N: number of sampled host species in each locality. The map was prepared using ArcGIS 10 (Arc Geographic Information System).

According to PCR-based testing, 121 of 237 songbird individuals (overall prevalence 51.1%), belonging to 14 families, 20 genera and 30 species were positive for *Plasmodium*, *Haemoproteus* and *Leucocytozoon* species (Table 1). Species of the Passeridae and Sylviidae were the most prevalently infected (44.6% and 19.0%, respectively). Prevalence of haemosporidian infections in the sampled localities varied from 32.2% (Khuzestan) to 2.5% (Hormozgan) (Figure 1).

### 2.2. Lineage Diversity

In total, 55 different haemosporidian parasite lineages were detected, of which 8 *Plasmodium* lineages (14.5% of all reported lineages), 35 *Haemoproteus* (63.6%) and 12 *Leucocytozoon* (21.8%). The highest lineage diversity was observed in birds of the Sylviidae and Passeridae families, with 21 and 16 different lineages found, respectively. Information about 15 novel lineages and the other identified lineages is given in Table 1. 

According to the MalAvi database, 13 lineages completely matched up with the lineages of previously described morphospecies: *Haemoproteus lanii* (RB1), *Haemoproteus attenuatus* (ROBIN1), *Haemoproteus passeris* (PADOM05), *Haemoproteus balmorali* (SFC1), *Haemoproteus parabelopolskyi* (SYAT01 and SYAT07), *Haemoproteus palloris* (WW1), *Haemoproteus belopolskyi* (ARW1 and MW1), *Haemoproteus nucleocondensus* (GRW01), *Haemoproteus sanguinis* (BUL1), *Plasmodium relictum* (GRW04 and SGS1) (Table 1). We identified 19 new host records for 21 previously known lineages; these records are shown in Table 1.

### 2.3. Co-Infection

We detected 13 co-infections of *Haemoproteus* spp. and *Leucocytozoon* spp. (Table 2). Co-infection of *Haemoproteus* spp. and *Plasmodium* spp. or *Plasmodium* spp. and *Leucocytozoon* spp. was not observed. Based on Pearson correlation, the probabilities of co-infection by *Haemoproteus* and *Leucocytozoon* parasites were not correlated (sig = 0.10, correlation was considered significant at the <0.01 level), but were negatively correlated with the occurrence of *Plasmodium* infection (sig = 0.00). We also identified 16 unresolved co-infections (unresolved double peaks) of parasites belonging to three haemosporidian genera, of which 7 belonged to *Haemoproteus* spp. lineages, 8 to *Leucocytozoon* spp. lineages and 1 to *Plasmodium* spp. lineages; these infections were excluded from further analysis and discussion.

## 3. Discussion

The key result of this study is the determination of the high prevalence and genetic diversity of avian haemosporidian parasite lineages in southern Iran, a formerly non-studied area in this regard. The studied localities cover a wide geographical area, including six provinces located in the Khuzestan province in the southwest, to Sistan and Baluchistan and South Khorasan provinces in the southeast and east of Iran (Figure 1). This study shows a high prevalence of haemosporidian parasites infecting wild Passeriformes birds, detected in over half of the examined bird species (Table 1). Briefly, these pathogens were detected in 73.3% of the examined bird species (N = 30/41), which belonged to 69% of the examined genera (N = 20/29) and 68.4% of the examined families (N = 14/20). Among all examined birds, the species of Passeridae (44.6% prevalence) and Sylviidae (19.0%) were the most prevalently infected. One-way ANOVA with post-hoc tests showed that infection prevalence in birds of the examined families was significantly different for parasites of the *Haemoproteus* and *Leucocytozoon* genera (sig = *0.01* and sig = *0.00,* respectively, *p* < 0.05), but was not significantly different for the parasites of the *Plasmodium* genus. 

The highest haemosporidian parasite infection prevalence was observed in Khuzestan (32.2%) and Bushehr (19.8%) provinces (Figure 1). The studied sites in these provinces were near rivers or marshes. According to previous studies [34,35], the availability of water bodies and a high humidity can increase the prevalence of haemosporidians due to a greater abundance of blood-sucking dipteran vectors. It is thus suggested to investigate the correlation between these environmental factors and the prevalence of haemosporidians in these regions in future studies. 

Fifty-five haemosporidian parasite lineages were detected. Fifteen lineages were new, being previously unknown parasite lineages worldwide (Table 1); among them, the lineages of *Haemoproteus* spp. (53.3% of all new lineages) and *Leucocytozoon* spp. (40%) predominated. New *Plasmodium* spp. lineages were rare (6.6% of new lineages). Interestingly, about 40% of the new lineages were found in White-eared Bulbul (*Pycnonotus leucotis*). This bird species is resident in southern parts of Iran [33], indicating that local transmission of the reported parasites occurs in Iran. This bird species deserves attention as a convenient model organism for research aiming to a better understanding of the local epidemiology of avian haemosporidiosis.

Thirty-five lineages were identified as belonging to *Haemoproteus* (63.6% of all reported lineages). The most prevalent were the lineages PADOM05, PAHIS2, CCF2, hHIP2 and SW4. The PADOM05 lineage belongs to *Haemoproteus passeris* [36], and its presence and high prevalence in Iran is congruent with a previous study [31]. This parasite has been reported in all zoogeographical regions except for the Antarctic [1] and is common around the Mediterranean Sea and in Central Europe [37]. The lineage PADOM05 was reported in Dead Sea sparrow (*Passer moabiticus)* and house sparrow (*Passer domesticus*) from the Eastern Mediterranean for the first time by Martinsen et al. [36,38]. The PADOM05 lineage was also reported in previous studies on birds of the Acrocephalidae, Sylviidae, Passeridae and Motacillidae families [31,36,38,39,40,41,42,43,44,45,46]. However, it remains unclear if birds other than species of the Passeridae are competent hosts for this parasite, because the PCR-based reports were not document by the presence of *H. passeris* gametocytes in the circulation. No study has reported this parasite species in non-passerine hosts according to the MalAvi database. Common nightingale *(Luscinia megarhynchos)*, Black redstart (*Phoenicurus ochruros)*, Yellow-throated sparrow *(Gymnoris xanthocollis)*, Grey hypocolius *(Hypocolius ampelinus)* and Willow warbler *(Phylloscopus trochilus)*, were recorded as new host species of the PADOM05 lineage in this study (Table 1). However, we also did not report gametocytes of *H. passeris* in these birds, and these hosts might be non-competent hosts for this parasite as well. Additional microscopic studies are needed to answer this question. 

To date, the species identity of the lineages PAHIS2, CCF2, HIP2 and SW4 remains unclear. Some of them might belong to new parasite species, and further microscopic examinations are needed to answer this question. The lineage PAHIS2 was reported in previous studies in house sparrow and Spanish sparrow (*Passer hispaniolensis*) [31,41,44]. The Eurasian blackcap (*Sylvia atricapilla)*, the willow warbler and the purple sunbird (*Cinnyris asiaticus*) were recorded as new host species for this lineage in this study. In previous studies, the CCF2 lineage was reported in birds of the Fringillidae, Paridae, Turdidae, Muscicapidae, Emberizidae and Hirundinidae families [41,46,47,48,49,50,51]. In this study, the lineage CCF2 was detected in white-eared bulbul*,* house sparrow and purple sunbird for the first time. The HIP2 lineage was reported only in the Eastern olivaceous warbler (*Iduna pallida*) and Booted warbler (*Iduna caligata*) of the Acrocephalidae family in previous studies [31,47,51]. The house sparrow was recorded as new host species for the HIP2 lineage in this study. Previously, the SW4 lineage was reported in the paddyfield warbler (*Acrocephalus agricola*) and Sedge warbler (*Acrocephalus schoenobaenus*) [52,53]. The great reed warbler (*Acrocephalus arundinaceus*) and house sparrow were recorded as new host species for this lineage in the current study. The other lineages of *Haemoproteus* spp. that were detected only in one host species were: PAHIS1, GRW01, ROBIN1, SFC1, SYHOR01, LWT1, SYAT01, SYAT07, RB1, BUL1, ARW1, MW1, MW3, PADOM03 and WW1 (Table 1). 

The second most prevalent haemosporidian parasites were species of *Leucocytozoon*, with 12 identified lineages, and 5 of them were new lineages (21.8% of all reported lineages). The lineage RS4 was the most prevalent lineage of *Leucocytozoon* spp., detected in different host species of different families. The species identity of this parasite remains unclear. The lineage RS4 was recorded in the common redstart (*Phoenicurus phoenicurus*), Eurasian blackcap, common whitethroat (*Sylvia communis*), berthelot's pipit (*Anthus berthelotii*), bluethroat (*Luscinia svecica*), forest fody (*Foudia omissa*), African stonechat (*Saxicola torquata*) in previous studies [51,52,54,55,56]. The house sparrow (*Passer domesticus*) and the yellow-throated sparrow were recorded as new host species for this lineage in this study. The other detected this genus lineages, which have been found in only one host species, were SYBOR07, SFC8, SYAT22, SYCON05, PARUS20 and AFR214 (Table 1).

Eight lineages of *Plasmodium* sp. were detected, and one lineage (LUME4, GenBank accession number MT925922) was new. The lineages LK05 and GRW04 were detected in different host species of different families. The LK05 lineage was detected in birds of four different families (Muscicapidae, Scotocercidae, Fringillidae and Sylviidae), while it was reported in species of the Falconidae, Motacillidae and Fringillidae previously [54,57,58,59]. Variable wheatear (*Oenanthe picata*), lesser whitethroat (*Sylvia curruca*), streaked scrub warbler (*Scotocerca inquieta*), European golden finch (*Carduelis carduelis*) are new host species for this malaria parasite lineage. The report of the same *Plasmodium* lineages in birds of different families was expected because avian malaria parasites often present broad vertebrate-host specificity and can complete their life cycle in birds belonging to different families and even orders [1,2,4]. 

The lineages GRW04, GRW11, LZFUS01 and SGS1 belong to the cosmopolitan malaria parasite, *Plasmodium relictum*; this parasite species is of global distribution and parasitizes many bird species [1]. The GRW04 and SGS1 lineages of *P. relictum,* which were detected in this study, have a widespread distribution and are virulent in many bird species [60,61]. According to the MalAvi database, the GRW04 and SGS1 lineages have been found in birds of 11 orders and in 21 families of Passeriformes. These two parasites are often virulent in various species of avian hosts [61]. In this study, the GRW04 lineage was detected in birds of three different families: Sylviidae (menetries's warbler *Sylvia mystacea*), Passeridae (house sparrow) and Pycnonotidae (white-eared bulbul), and the SGS1 was detected in Muscicapidae (rufous-tailed scrub robin *Cercotrichas galactotes*) and Passeridae (house sparrow). It is interesting to note that the report of both these malaria lineages in non-migrating house sparrow at same study sites indicate the local transmission in sympatry, providing opportunities to study the influence of this co-infection in birds during natural infection in the future. The lineages SYBOR02, SYBOR10, PADOM02 and PBPIP1 were found in only one host species (Table 1).

We detected 13 co-infections (9.8% of all infected individuals) of *Haemoproteus* spp. and *Leucocytozoon* spp. lineages (Table 2). These co-infections were detected in five different host species. The lineages RS4 and SYAT22 of *Leucocytozoon* spp. were present most often in co-infections. The lineage RS4 was detected in five co-infections in two different host species (common whitethroat and house sparrow); it was present together with the lineages SYNIS2, CWT2, PAHIS2, PADOM05 and HIP2 (*Haemoproteus* spp.). The lineage SYAT22 (*Leucocytozoon* spp.) was detected in two co-infections in the Eurasian blackcap; it was present together with the PAHIS2 and SYAT07 (*Haemoproteus* spp.) lineages. 

In this study, a Pearson correlation test of co-infection by *Haemoproteus* and *Leucocytozoon* parasites was not significant. Van Rooyen et al. [61] reported the presence of 82% co-infection by parasites belonging to different genera in great tits. They found more lineage diversity of *Haemoproteus* during co-infections with *Leucocytozoon* parasites and speculated that the parasites can more easily become established in hosts whose immune system is weakened by prior infections. However, this hypothesises cannot be supported by the data collected in this study, and further studies are needed to clarify this issue. For unclear reasons, we did not find any co-infection of *Plasmodium* and *Leucocytozoon* or *Haemoproteus* and *Plasmodium* parasites, while co-infections of these parasites are common in wildlife [5,16,62,63,64,65]. This might be due to preferable amplification of the DNA of *Haemoproteus* or *Plasmodium* parasite lineages in our samples during co-infections [66]. This study shows that the application of more sensitive diagnostic methods (parasite genus-specific primers or/and multiplex PCR essays) is essential to clarify this issue in the future [67,68].

PCR-based methods often underestimate the diversity of haemosporidians during co-existence of genetically similar parasite lineages in co-infections [66,69,70]. Available PCR assays often preferentially amplify the DNA of one species in the same individual bird co-infected with two different parasites of the *Plasmodium* and *Haemoproteus* genera [66,71]. On the other hand, the parasite lineages presented in a sample and the DNA quantity and quality may affect a study results [69,72,73]. Using both PCR-based and microscopic methods in parallel can increase the detectability of haemosporidian infections, especially co-infections of different parasites in wild bird populations [74,75] and is worth application in wildlife haemosporidian research. We excluded 16 parasite DNA sequences, which were identified as unresolved co-infections from our analysis because their identification was impossible through the PCR-based method.

One-way ANOVA test results showed that the most prevalently infected birds were species of the Passeridae, and the biggest lineage diversity was seen in species of the Sylviidae. Valkiūnas [1] reported a high prevalence of haemosporidian parasites in birds of the Sylviidae family in Europe. In our study, four species of *Sylvia* were most prevalently infected with haemosporidian parasites. These are Hume's whitethroat (*Sylvia althaea*) (5.8% prevalence), barred warbler (*Sylvia nisoria*) 1.6%, Eurasian blackcap 3.3% and common whitethroat 6.6%. More than half of individuals of these four species showed double infection with *Haemoproteus* and *Leucocytozoon* parasites, and some of them were unresolved co-infections and were removed from analysis. On the other hand, the co-infection rate in the house sparrow was only 9.5%, despite the big sample size (80 specimens). The prevalence of *Haemoproteus* infection in the house sparrow was 15.2%, but only 5% of house sparrows were infected with both *Haemoproteus* and *Leucocytozoon* parasites. It seems that the house sparrow can be partially resistant to the haemoproteids normally developing in other songbirds [37]. According to Loiseau et al. [76], specific MHC (major histocompatibility complex) alleles, which are associated with susceptibility to *Plasmodium* spp. in a population of house sparrows, can confer resistance to a co-infection with *Haemoproteus* spp. Based on the MalAvi database, 46 lineages of *Plasmodium* spp., 28 lineages of *Haemoproteus* spp. and only 6 lineages of *Leucocytozoon* spp. were reported in house sparrows. Many more lineages (47 lineages of *Haemoproteus* spp., 14 of *Leucocytozoon* spp. and 9 of *Plasmodium* spp.) were reported in the above-mentioned species of *Sylvia* around the world. The available data might indicate a relatively higher natural resistance in house sparrows to these infections in comparison to species of the genus *Sylvia*, which probably are more susceptible to *Haemoproteus* and *Leucocytozoon* infections. These results might also indicate that house sparrows have higher resistance to *Leucocytozoon* parasites. However, this issue still needs to be studied more extensively. Defence strategies during infection vary among different host species; therefore, some bird species are more resistant to same parasites than others [77]. Palinauskas et al. [78] showed that starlings were resistant to single and double experimental malaria infections, but many other passeriform bird species were susceptible. Van Rooyen et al. [62] showed that great tits (*Parus major)* were more resistant to *Leucocytozoon* parasites than to *Haemoproteus* and *Plasmodium* parasites. In addition to host immune defence, within-host competition between parasites, patterns of local transmission and availability of vectors can affect the occurrence of co-infection in certain avian hosts [62,77,79].

About half of the co-infections were observed in Khuzestan (Karkheh protected area) (Table 2), indicating the high rate of transmission at this study site, probably due to a high abundance of vectors [1]. Climatic and habitat conditions are important factors affecting the activity and abundance of vectors [80,81]; this needs further investigation in this area. 

Many studies reported co-infections with haemosporidian parasites belonging to different genera. Valkiūnas et al. [63] found that 86.0% of the examined hawfinches *Coccothraustes coccothraustes* were co-infected with haemosporidian parasites belonging to different genera. Marzal et al. [5] detected 16% of double infections with *Haemoproteus* spp. and *Plasmodium* spp. lineages in house martins. Zehtindjiev et al. [82] reported the death of great reed warblers after co-infection with two *Plasmodium* species. Van Rooyen et al. [62] reported co-infection in 81.5% of infected great tits, including various combinations of *Plasmodium* or *Haemoproteus* and *Leucocytozoon*. Schoener et al. [83] found co-infection of *Plasmodium* lineages in blackbirds (*Turdus merula*) and song thrushes (*Turdus philomelos*) in New Zealand. Some studies have shown that co-infection with parasites of different haemosporidian genera can increase pathogen virulence and mortality in the host populations [5,84,85]. Due to short-term field study, we likely missed the acute primary infection stage during haemosporidian parasite co-infections, and further studies are needed for a better understanding of the mortality and epidemiology of such infections. 

We detected 116 (48.9%) haemosporidian-free individuals in 237 sampled passerines. These uninfected individuals were mostly from bird groups with less than five sampled individuals that belonged to 11 families, 12 genera and 13 species (Table 1). 

Along with the results of this study (Table 1), 33 formerly known lineages were reported in resident bird species in Iran (based on the MalAvi database). We reported for the first time haemosporidian infections in a non-migrating passerine species in the southern parts of Iran, the white-eared bulbul. This bird is the resident species, which is common in palm trees and gardens in regions in southern parts of Iran as well as in Iraq and Syria [86]. Half of the examined individuals of white-eared bulbul were infected by haemosporidian parasites, and 7.8% of the co-infections were also detected in this species (Table 2). In all, nine lineages were found in this species, of which six were new. The lineages CCF2, BUL1 and GRW04 were detected in this species, indicating the local transmission of these parasites. These data are in accordance with a report of the GRW04 and pSGS1 lineages in the non-migratory house sparrow in Iran (Table 1). Both these bird species can be recommended as a good model of avian hosts for the investigation of locally transmitted haemosporidian infections and of haemosporidiosis epidemiology in Iran. 

## 4. Materials and Methods

### 4.1. Collection of the Samples

Collection of the samples was performed in south and southeast of Iran from 2017 (February to July) to 2018 (April to July). Birds were sampled at eight different sites in six provinces using mist nets, as shown in Figure 1. Data about the sampled birds belonging to different families, genera and species at each site are shown in Table 1. 

In total, blood samples from 237 songbirds belonging to 29 genera, 41 species and 20 families were collected. Bird species were identified according to Svensson [86]. A drop of blood was taken from each bird by puncturing the brachial vein with sterile insulin needles. About 30 µL of blood was collected in heparinized microcapillaries and stored in 1.5 mL microcentrifuge tubes containing about 700 μL of Queens's buffer at ambient temperature while in the field, and then preserved at −20 °C in the laboratory. All birds were released after sampling, and all efforts were made to minimize their suffering. 

### 4.2. DNA Extraction, PCR and Sequencing 

Total genomic DNA extraction was conducted using the standard salt extraction method described by Bruford et al. [87]. All collected samples were processed using a nested PCR protocol [88] targeting a 479-base-pair fragment of the *cytb* gene of *Haemoproteus*, *Plasmodium* and *Leucocytozoon* species. Initial PCR was performed with the primers HaemFN1/HaemR3N which amplified the DNA of parasites belonging to all three genera, i.e., *Plasmodium*, *Haemoproteus* and *Leucocytozoon*. The PCR reaction was performed in 25 µL total volume containing 1.5 µL MgCl2 (25 mM), 2.5 µL GeneAmp 10× PCR Buffer, 2.5 µL dNTP (1.25 mM), 0.1 µL AmpliTaq DNA polymerase (5 U/µL), 0.6 mM of each primer, 15.4 µL of double distilled H2O and 1 µL of diluted total genomic DNA template (25 ng/µL), per reaction; subsequent PCR was performed with the primers HaemF/HaemR2 for *Plasmodium* and *Haemoproteus* and HaemFL/HaemR2L for *Leucocytozoon* parasites. The nested PCR reaction was also performed in 25 µL total volume containing 2 µL of the first PCR product and the same concentration of the first PCR reagents. To check if the PCRs had been successful, 2.5 µL of the final PCR product was run on a 2% agarose gel. Negative (ultrapure water) and positive (microscopy-positive samples) controls were used to verify the results of every PCR run. All PCR products were purified and sequenced by Microsynth AG (Balgach, Switzerland). 

The sequences of 479 base pairs of the *cytb* gene were edited and aligned in BioEdit [89], then they were compared with lineages available in the MalAvi Public Database (MalAvi http://mbio-serv2.mbioekol.lu.se/Malavi/, accessed on 1 May 2021) and National Center for Biotechnology Information (NCBI) (https://blast.ncbi.nlm.nih.gov/Blast.cgi, accessed on 1 May 2021). The sequences possessing at least one different nucleotide were considered as new lineages and were named according to the MalAvi nomenclature. Sequences with double peaks in the chromatograms were identified as unresolved co-infections. New sequences and unresolved co-infections were sequenced from the 3´end using HaemR2 (*Haemoproteus*-*Plasmodium* spp.-positive samples) or HaemR2L (*Leucocytozoon* spp.-positive samples) for the sequence validation, and unresolved co-infections were excluded from the analysis. All obtained DNA sequences were deposited in GenBank (accession numbers MT925798-MT925931, see Table 1). 

### 4.3. Statistical Analysis

Statistical analysis was performed using Statistical Package for the Social Sciences (SPSS) for windows, version 14.0 (SPSS Inc., Chicago, IL, USA). To assess prevalence differences among bird species and birds of different families as well as correlation of simultaneous presence of multiple parasite genera in a bird host, one-way ANOVA and Pearson correlation tests were used, as reported in the text. Only data on birds belonging to five most extensively sampled families (Acrocephalidae, Muscicapidae, Passeridae, Pycnonotidae, Sylviidae), were incorporated in the statistical analysis, aiming to a comparative prevalence analysis. In each bird family, over 14 individuals were sampled (Table 1).

## 5. Conclusions

This study revealed a high prevalence and diversity of haemosporidian parasites in passerines in Iran and provides extended information about the distribution of these infections in southern Asia. In all, 93 parasite lineages were reported in birds in Iran. Notably, 36 lineages, including 15 new lineages, were found in the resident (non-migrating) bird species, indicating active local transmission of haemosporidiosis and calling for further research on these blood parasites. It is important to note that the invasive lineages SGS1 and GRW4 of *P. relictum*, which cause virulent avian malaria, are present and transmitted in sympatry in Iran. The common presence of co-infections, which are often more virulent than single infections, shows that haemosporidians might be important for wild bird health in this region. Further studies are needed to understand the epidemiological patterns of avian haemosporidiosis and the role of these diseases in natural ecosystems. This study determined the most convenient avian model hosts and common haemosporidian parasites lineages for such research in southern Iran.

## Figures and Tables

**Table 1:** Avian haemosporidian parasite diversity and prevalence in birds sampled in southern Iran. Parasite species, cytochrome *b* lineage GenBank accessions and migration status of bird species are shown. Newly detected lineages are given in bold.

Host Family and Species	Study Site	Migration Status	N^a^	Parasite Species	Lineage (GenBank Accession)	Molecular Prevalence of Lineage (%) ^b^
**Acrocephalidae**						
*Acrocephalus arundinaceus* ^c^	A, B	P	6	*Leucocytozoon* sp.	AFR214 (MT925903)	1 (16.7)
				*Haemoproteus* sp.	SW4 (MT925861)	1 (16.7)
				*Haemoproteus nucleocondensus*	GRW01 (MT925867)	1 (16.7)
				*Plasmodium* sp.	SYBOR10 (MT925889)	1 (16.7)
*Acrocephalus dumetorum*	G	B P	1	0	0	0
*Acrocephalus palustris*	B	B P	4	*Haemoproteus belopolskyi*	ARW1 (MT925863)	1 (25.0)
				*Haemoproteus* sp.	MW3 (MT925871)	1 (25.0)
*Acrocephalus scirpaceus*	B	B	7	*Haemoproteus belopolskyi*	ARW1 (MT925863)	1 (14.3)
				*Haemoproteus belopolskyi*	MW1 (MT925870)	1 (14.3)
*Iduna pallida*	A, B	B	4	*Haemoproteus* sp.	HIP2 (MT925849)	2 (50.0)
**Alaudidae**						
*Calandrella* *brachydactyla*	G	B	2	*Leucocytozoon* sp.	WW6 (MT925894)	1 (50.0)
*Galerida cristata*	H	R	1	0	0	0
**Emberizidae**						
*Emberiza cineracea*	B	B	1	0	0	0
*Emberiza melanocephala*	A	B P	1	0	0	0
**Estrildidae**						
*Lonchura malabarica*	C	R	1	0	0	0
**Fringillidae**						
*Carduelis carduelis* ^c^	H	R	2	*Plasmodium* sp.	LK05 (MT925884)	1 (50.0)
**Hypocoliidae**						
*Hypocolius ampelinus* ^c^	A	W B	1	*Haemoproteus passeris*	PADOM05 (MT925798)	1 (100)
**Laniidae**						
*Lanius isabellinus* ^c^	F	W B P	1	*Haemoproteus lanii*	RB1 (MT925874)	1 (100)
**Leiothrichidae**						
*Argya caudata*	A, E	R	3	*Haemoproteus* sp.	**ARGCAU1** (MT925919)	2 (66.7)
**Locustellidae**						
*Locustella luscinioides*	B	P	1	0	0	0
**Motacillidae**						
*Anthus similies*	C	W B	1	0	0	0
*Motacilla alba*	H	R	1	0	0	0
**Muscicapidae**						
*Cercotrichas galactotes*	A	B	2	*Plasmodium relictum*	SGS1 (MT925890)	1 (50.0)
*Irania gutturalis*	F	B	1	0	0	0
*Luscinia megarhynchos* ^c^	C, F, G	B	8	*Haemoproteus attenuatus*	ROBIN1 (MT925844)	2 (25.0)
				*Haemoproteus passeris*	PADOM05 (MT925798)	1 (12.5)
				*Plasmodium* sp.	**LUME4** (MT925922)	1 (12.5)
				*Plasmodium* sp.	SYBOR02 (MT925888)	1 (12.5)
*Muscicapa striata*	G	B P	1	*Haemoproteus balmorali*	SFC1 (MT925875)	1 (100)
*Oenanthe picata* ^c^	H	W B P	1	*Plasmodium* sp.	LK05 (MT925884)	1 (100)
*Phoenicurus ochruros* ^c^	A	W B	1	*Haemoproteus passeris*	PADOM05 (MT925798)	1 (100)
**Nectariniidae**						
*Cinnyris asiaticus* ^c^	D	R	4	*Haemoproteus* sp.	PAHIS2 (MT925825)	1 (25.0)
				*Haemoproteus* sp.	CCF2 (MT925853)	1 (25.0)
**Oriolidae**						
*Oriolus oriolus*	H	P	1	0	0	0
**Passeridae**						
*Gymnoris xanthocollis* ^c^	A	B	12	*Haemoproteus* sp.	**GYMXAN1** (MT925915)	2 (16.7)
				*Haemoproteus* sp.	**GYMXAN2** (MT925917)	2 (16.7)
				*Haemoproteus passeris*	PADOM05 (MT925798)	4 (33.3)
				*Leucocytozoon* sp.	RS4 (MT925895)	1 (8.3)
*Passer domesticus* ^c^	A,B,D,E,H	R	80	*Haemoproteus* sp.	PAHIS1 (MT925822)	2 (2.5)
				*Haemoproteus passeris*	PADOM05 (MT925798)	15 (18.7)
				*Haemoproteus* sp.	PAHIS2 (MT925825)	12 (15.0)
				*Haemoproteus* sp.	HIP2 (MT925849)	2 (2.5)
				*Haemoproteus* sp.	**PADOM34 **(MT925921)	1 (1.2)
				*Haemoproteus* sp.	PADOM03 (MT925868)	2 (2.5)
				*Haemoproteus* sp.	CCF2 (MT925853)	1 (1.2)
				*Plasmodium relictum*	SGS1 (MT925890)	1 (1.2)
				*Plasmodium relictum*	GRW04 (MT925877)	2 (2.5)
				*Plasmodium* sp.	PADOM02 (MT925882)	1 (1.2)
				*Leucocytozoon* sp.	**PADOM35 **(MT925930)	1 (1.2)
				*Leucocytozoon* sp.	RS4 (MT925895)	4 (5.0)
*Passer hispaniolensis*	A	W P	1	*Haemoproteus passeris*	PADOM05 (MT925798)	1 (100)
*Passer moabiticus* ^c^	A, B	R	7	*Haemoproteus* sp.	SW4 (MT925861)	1 (14.3)
				*Haemoproteus* sp.	PAHIS1 (MT925822)	1 (14.3)
				*Plasmodium* sp.	PADOM02 (MT925882)	1 (14.3)
				*Plasmodium* sp.	PBPIP1 (MT925881)	1 (14.3)
*Passer montanus*	H	R	4	0	0	0
**Phylloscopidae**						
*Phylloscopus collybita*	C	W P	2	0	0	0
*Phylloscopus trochilus* ^c^	A, B	P	5	*Haemoproteus* sp.	PAHIS2 (MT925825)	1 (20.0)
				*Haemoproteus passeris*	PADOM05 (MT925798)	1 (20.0)
				*Haemoproteus palloris*	WW1 (MT925860)	1 (20.0)
**Pycnonotidae**						
*Pycnonotus leucotis* ^c^	A,D,E,F	R	24	*Haemoproteus* sp.	**PYCLEU2 **(MT925913)	1 (4.2)
				*Haemoproteus* sp.	CCF2 (MT925853)	1 (4.2)
				*Haemoproteus sanguinis*	BUL1 (MT925876)	1 (4.2)
				*Plasmodium* sp.	**LUME4 **(MT925922)	2 (8.3)
				*Plasmodium relictum*	GRW04 (MT925877)	1 (4.2)
				*Leucocytozoon* sp.	**PYCLEU1 **(MT925926)	1 (4.2)
				*Leucocytozoon* sp.	**PYCLEU3** (MT925925)	1 (4.2)
				*Leucocytozoon* sp.	**PYCLEU4** (MT925929)	1 (4.2)
				*Leucocytozoon* sp.	**PYCLEU5** (MT925931)	1 (4.2)
**Scotocercidae**						
*Scotocerca inquieta* ^c^	H	R	1	*Plasmodium* sp.	LK05 (MT925884)	1 (100)
**Sittidae**						
*Sitta tephronata* ^c^	F,G,H	R	5	*Haemoproteus* sp.	**SITTEP02 **(MT925909)	1 (20.0)
				*Haemoproteus* sp.	**SITTEP03** (MT925910)	2 (40.0)
				*Leucocytozoon* sp.	PARUS20 (MT925904)	1 (20.0)
**Sturnidae**						
*Sturnus roseus*	H	B	1	0	0	0
**Sylviidae**						
*Sylvia althaea* ^c^	F,G,H	B	9	*Haemoproteus* sp.	SYHOR01 (MT925840)	4 (44.4)
				*Haemoproteus* sp.	**SYLALT01 **(MT925912)	1 (11.1)
				*Haemoproteus* sp.	CURCUR01 (MT925872)	1 (11.1)
				*Haemoproteus* sp.	LWT1 (MT925873)	1 (11.1)
				*Leucocytozoon* sp.	SYBOR07 (MT925892)	1 (11.1)
				*Leucocytozoon* sp.	SFC8 (MT925893)	1 (11.1)
				*Leucocytozoon* sp.	**SYNIS4 **(MT925927)	1 (11.1)
*Sylvia atricapilla* ^c^	A	P	7	*Haemoproteus* sp.	PAHIS2 (MT925825)	1 (14.3)
				*Haemoproteus parabelopolskyi*	SYAT01 (MT925846)	1 (14.3)
				*Haemoproteus parabelopolskyi*	SYAT07 (MT925847)	2 (28.6)
				*Leucocytozoon* sp.	SYAT22 (MT925906)	3 (42.8)
*Sylvia communis* ^c^	A	P	12	*Haemoproteus* sp.	SYNIS2 (MT925859)	1 (8.3)
				*Haemoproteus* sp.	CWT2 (MT925856)	2 (16.7)
				*Haemoproteus* sp.	CWT3 (MT925865)	2 (16.7)
				*Haemoproteus* sp.	CWT7 (MT925858)	1 (8.3)
				*Leucocytozoon* sp.	RS4 (MT925895)	3 (25.0)
				*Leucocytozoon* sp.	SYCON05 (MT925905)	1 (8.3)
*Sylvia curruca* ^c^	A,B,C	P	4	*Plasmodium* sp.	LK05 (MT925884)	1 (25.0)
*Sylvia mystacea*	A	P	1	*Plasmodium relictum*	GRW04 (MT925877)	1 (100)
*Sylvia nisoria*	A,B	P	4	*Haemoproteus* sp.	**PYCLEU2 **(MT925913)	1 (25.0)
				*Leucocytozoon* sp.	**SYNIS4** (MT925927)	1 (25.0)
20 families, 29 genera, 41 species	8 sites	4 migration status birds	237	13 parasite species	55 lineages	Overall prevalence 121 (51.1)

Study sites: A, Khuzestan province; B, Bushehr province; C, Hormozgan province; D, Rask (Sistan and Baluchistan province); E, Saravan (Sistan and Baluchistan province); F, Bam (Kerman province); G, Baft (Kerman province); H, South Khorasan province. Migration status: W = Wintering, B = Breeding, R = Resident, P = Passengers. N^a^: Number of sampled individuals. Superscript b (^b^): Number of detected infections, followed in parentheses by the percentage. Superscript c (^c^): New hosts reports of corresponding parasite lineages.

**Table 2:** Co-infection of *Haemoproteus* spp. and *Leucocytozoon* spp. lineages in birds sampled in southern Iran. Cytochrome *b* lineage GenBank accession numbers are given in parentheses.

Host Species	*Haemoproteus* Lineage (GenBank Accession)	*Leucocytozoon* Lineage (GenBank Accession)	N^a^	Study Site ^b^
*Sylvia althaea*	SYHOR01 (MT925840)	SYBOR07 (MT925892)	1	F
*S. althaea*	LWT1 (MT925873)	SFC8 (MT925893)	1	F
*S. althaea*	CURCUR01 (MT925872)	SYNIS4 (MT925927)	1	H
*S. atricapilla*	SYAT07 (MT925847)	SYAT22 (MT925906)	2	A
*S. atricapilla*	PAHIS2 (MT925825)	SYAT22 (MT925906)	1	A
*S. communis*	SYNIS2 (MT925859)	RS4 (MT925895)	1	A
*S. communis*	CWT2 (MT925856)	RS4 (MT925895)	1	A
*Passer domesticus*	PAHIS2 (MT925825)	RS4 (MT925895)	1	A
*P. domesticus*	PADOM05 (MT925798)	RS4 (MT925895)	2	E
*P. domesticus*	HIP2 (MT925849)	RS4 (MT925895)	1	E
*Pycnonotus leucotis*	BUL1 (MT925876)	PYCLEU5 (MT925931)	1	C
Total	10 lineages	6 lineages	13	5 sites

N^a^: Number of co-infections identified. Superscript b (^b^): Study site abbreviations are as in Table 1.

## Data Availability

The amplified sequences of the parasite lineages (mitochondrial *cytb* gene) were deposited in GenBank (Accession numbers MT925798-MT925931).
